# Isobacachalcone induces autophagy and improves the outcome of immunogenic chemotherapy

**DOI:** 10.1038/s41419-020-03226-x

**Published:** 2020-11-26

**Authors:** Qi Wu, Ai-Ling Tian, Sylvère Durand, Fanny Aprahamian, Nitharsshini Nirmalathasan, Wei Xie, Peng Liu, Liwei Zhao, Shuai Zhang, Hui Pan, Didac Carmona-Gutierrez, Frank Madeo, Yi Tu, Oliver Kepp, Guido Kroemer

**Affiliations:** 1 Department of Breast and Thyroid Surgery, Renmin Hospital of Wuhan University, Wuhan, China; 2 Centre de Recherche des Cordeliers, Equipe labellisée par la Ligue contre le cancer, Université de Paris, Sorbonne Université, Inserm U1138, Institut Universitaire de France, Paris, France; 3 Metabolomics and Cell Biology Platforms, Gustave Roussy Cancer Center, Université Paris Saclay, Villejuif, France; 4 Faculty of Medicine, Université Paris Saclay, Kremlin-Bicêtre, France; 5 Institute of Molecular Biosciences, NAWI Graz, University of Graz, Graz, Austria; 6 BioTechMed-Graz, Graz, Austria; 7 Field of Excellence BioHealth, University of Graz, Graz, Austria; 8 Suzhou Institute for Systems Medicine, Chinese Academy of Medical Sciences, Suzhou, China; 9 Pôle de Biologie, Hôpital Européen Georges Pompidou, AP-HP, Paris, France; 10 Karolinska Institutet, Department of Women’s and Children’s Health, Karolinska University Hospital, Stockholm, Sweden

**Keywords:** Immunosurveillance, Macroautophagy

## Abstract

A number of natural plant products have a long-standing history in both traditional and modern medical applications. Some secondary metabolites induce autophagy and mediate autophagy-dependent healthspan- and lifespan-extending effects in suitable mouse models. Here, we identified isobacachalcone (ISO) as a non-toxic inducer of autophagic flux that acts on human and mouse cells in vitro, as well as mouse organs in vivo. Mechanistically, ISO inhibits AKT as well as, downstream of AKT, the mechanistic target of rapamycin complex 1 (mTORC1), coupled to the activation of the pro-autophagic transcription factors EB (TFEB) and E3 (TFE3). Cells equipped with a constitutively active AKT mutant failed to activate autophagy. ISO also stimulated the AKT-repressible activation of all three arms of the unfolded stress response (UPR), including the PERK-dependent phosphorylation of eukaryotic initiation factor 2α (eIF2α). Knockout of TFEB and/or TFE3 blunted the UPR, while knockout of PERK or replacement of eIF2α by a non-phosphorylable mutant reduced TFEB/TFE3 activation and autophagy induced by ISO. This points to crosstalk between the UPR and autophagy. Of note, the administration of ISO to mice improved the efficacy of immunogenic anticancer chemotherapy. This effect relied on an improved T lymphocyte-dependent anticancer immune response and was lost upon constitutive AKT activation in, or deletion of the essential autophagy gene *Atg5* from, the malignant cells. In conclusion, ISO is a bioavailable autophagy inducer that warrants further preclinical characterization.

## Introduction

Macroautophagy (to which we herein refer as “autophagy”) is a unique cell biology phenomenon that leads to cytoplasmic vacuolization in response to nutrient deprivation as well as to a myriad of other cell stress-inducing conditions^1^. Portions of the cytoplasm are enveloped in two-membraned vesicles, the autophagosomes, which then fuse with lysosomes for the digestion of the autophagic cargo by hydrolases that operate at acidic pH^2,3^. Autophagy allows to mobilize the cell’s energy reserves by digestion of cytoplasmic macromolecules and even entire organelles to recover their building blocks, including amino acids, simple sugars, and free fatty acids^4^. In addition, autophagy allows for the selective degradation of superficial, damaged, or aged cellular components, including dysfunctional organelles and potentially pathogenic protein aggregates. Genetic stimulation of autophagy has potent antiaging properties, reducing the manifestation of age-associated diseases, including arteriosclerosis, cancer, and neurodegeneration^5–7^. Pharmacological induction of autophagy has similar broad healthspan and lifespan-extending effects, as shown in model organisms including yeast, nematodes, flies, and mice^8–11^.

Obviously, there is much interest in identifying novel autophagy inducers that operate at low levels of toxicity and mediate broad antiaging and pro-health effects. Chalcones belong to the chemical class of flavonoids and are contained in multiple plants that are reputed for their dietary virtues. Based on these considerations, we have set out in the past to identify autophagy-inducing chalcones. Among a homemade library of chalcones, we identified two different agents, namely, 4,4′-dimethoxychalcone (4,4′DMC)^12^ and its isomer 3,4-dimethoxychalcone (3,4-DMC)^13^ as potent autophagy inducers. Of note, both chalcones differ in their mode of action. While 4,4′DMC inhibits autophagy-suppressive GATA transcription factors^12,14^, 3,4-DMC acts through the activation of the two related pro-autophagic transcription factors EB (TFEB) and E3 (TFE3)^13^. Irrespective of this difference, both 4,4′DMC and 3,4-DMC reduce myocardial infarction in mice. Moreover, 4,4′DMC extended the lifespan of yeast, nematodes, and flies^12^, while 3,4-DMC enhanced anticancer immune responses in mice^13^. These preclinical data plead in favor of a potential medial utility for chalcones.

Driven by these considerations, we decided to identify additional pro-autophagic chalcones by screening another collection of agents. Here, we demonstrate that isobacachalcone (ISO) stimulates autophagic flux, delineate the molecular pathways involved in this effect, and suggest clinical utility for this chalcone as a stimulator of anticancer immunity in the context of immunogenic cell death (ICD)-inducing chemotherapy.

## Results

### Identification of ISO as an inducer of autophagic puncta

**Fig. 1:**
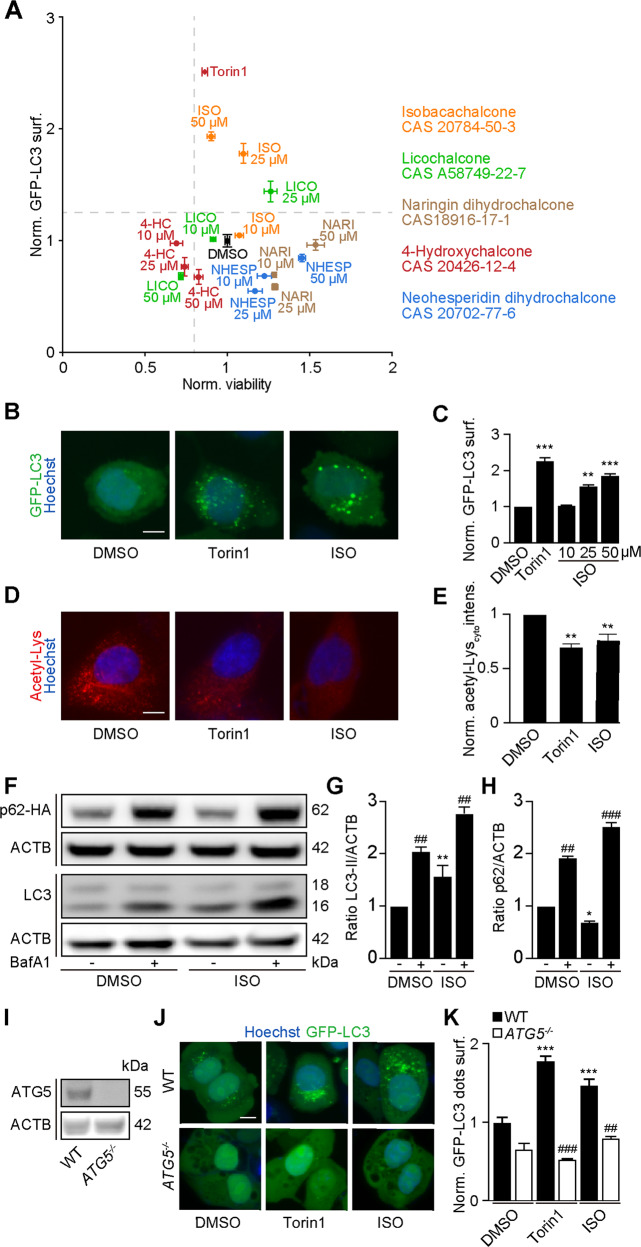
Isobacachalcone (ISO) is a candidate caloric restriction mimetic (CRM). **A** Human neuroglioma H4 cells stably expressing GFP-LC3 were treated with a selection of chalcones from the TargetMol library of flavonoids at the indicated concentrations. We compared the selected agents at different concentrations with the standard autophagy inducer torin 1 (300 nM), and identified conditions with significantly increased GFP-LC3 puncta formation (1.25 times of the vehicle control (DMSO)) and viability of at least 80% with respect to DMSO, as potent autophagy activation. **B**, **C** H4 cells stably expressing GFP-LC3 were treated with isobacachalcone (ISO) (10, 25, and 50 μM) for 6 h. Then the cells were fixed and imaged to assess the formation of GFP-LC3 puncta (**C**). Torin 1 (300 nM) was used as a prototypical autophagy inducer. Representative images are shown in (**B**). Scale bar equals 10 μm. Data are means ± SD of quadruplicates (^**^P < 0.01; ^***^P < 0.001 vs. DMSO/Ctr, Student’s *t* test). **D**, **E** U2OS cells were treated as described above, followed by the incubation with specific antibodies to block acetylated tubulin. Thereafter, immunofluorescence was conducted with antibodies against acetylated lysine residues and appropriate AlexaFluor-conjugated secondary antibodies. Representative images of lysine acetylation are shown in (**D**), and the decrease of acetylation in the cytoplasm was measured in (**E**). Scale bar equals 10 μm. Data are means ± SD of quadruplicates (^**^P < 0.01 vs. DMSO/Ctr, Student’s *t* test). **F**, **H** U2OS cells transfected with a plasmid expressing p62 protein fused with an HA tag (HA-p62) were treated with ISO (25 μM) in the presence or absence of bafilomycin A1 (Baf A1, 100 nM) for 6 h. SDS–PAGE and immunoblot were performed, band intensities of HA-p62 and β-actin (ATCB) were assessed, and the ratio (HA/ATCB) was calculated (**H**). In parallel samples, band intensities of LC3-II and ATCB were assessed, and their ratio (LC3-II/ATCB) was calculated (**G**). Data are means ± SD of three independent experiments (^*^P < 0.05, ^**^P < 0.01 vs. untreated control; ^##^*P* < 0.01, ^###^*P* < 0.001 vs. without Baf A1; Tukey’s multiple comparisons test). **I**, **K** Human osteosarcoma U2OS cell stably expressing GFP-LC3 either wild-type (WT) or ATG5 knockout (**I**) were treated with ISO (25 μM) or torin 1 (300 nM) for 6 h. The cells were fixed, imaged, and GFP-LC3 dots were quantified (**K**). Scale bar equals 10 μm. Data are means ± SD of quadruplicates (^***^P < 0.001 vs. untreated control; ^##^*P* < 0.01, ^###^*P* < 0.001 vs. WT; Tukey’s multiple comparisons test).

Human neuroglioma H4 cells stably transduced with a fusion protein containing green fluorescent protein (GFP) in the N- and microtubule-associated proteins 1A/1B light chain 3B (MAP1LC3B, best known as LC3) in the C-terminus (GFP-LC3) were cultured in the presence of each of the chalcones contained in the Polyphenolic Natural Compound Library from TargetMol, each used at three different concentrations (10, 25, and 50 µM). We found that ISO, (E)-1-[2,4-dihydroxy-3-(3-methyl-2-butenyl)-phenyl]-3-(4-hydroxyphenyl)-2-propen-1-one or (E)-4,2′,4′-trihydroxy-3′-prenylchalcone; 2′,4,4′-trihydroxy-3′-prenyl-transchalcone) consistently induced GFP-LC3 puncta at doses of 25 and 50 µM (Fig. 1A–C). This effect was coupled to a reduction in cytoplasmic protein acetylation that could be measured by immunofluorescence assays using antibodies that recognize acetylated lysine residues (Fig. 1D, E). ISO also induced the lipidation of LC3, measurable by immunoblot analyses (in which LC3 lipidation yields a band with higher electrophoretic mobility, i.e., LC3B or LC3-II) that was even observed in the presence of bafilomycin A1, an inhibitor of autophagosome-lysosome fusion, suggesting that ISO induces autophagic flux (Fig. 1F, G). Simultaneously, ISO reduced the abundance of hemagglutinin (HA)-tagged sequestosome 1 (SQSTM1, best known as p62) fusion protein transfected into the cells, again supporting the idea that ISO stimulates autophagic flux (Fig. 1F, H). In human osteosarcoma U2OS cells, ISO also induced GFP-LC3 puncta but failed to do so upon knockout of the essential autophagy gene *ATG5* (Fig. 1I–K), indicating that the formation of GFP-LC3 puncta is indeed coupled to autophagy. In sum, it appears that ISO is a chalcone endowed with autophagy-stimulatory properties.

### ISO induces autophagic puncta through the inhibition of AKT

**Fig. 2:**
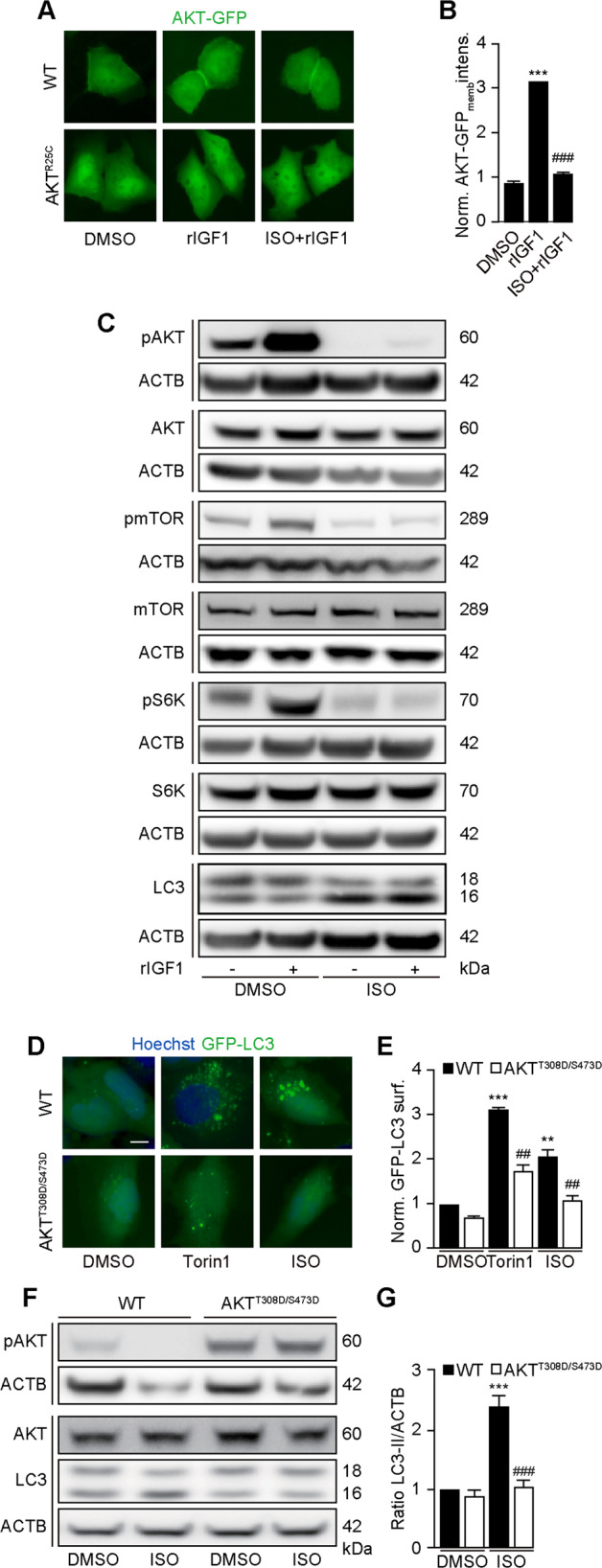
Inhibition of AKT phosphorylation is pivotal to ISO-induced autophagy. **A**, **B** Human osteosarcoma U2OS cells stably expressing GFP-AKT or GFP-AKT^R25C^ were treated serum-deprived overnight, then the cells were treated with recombinant IGF1 (rIGF1, 10 nM) or isobacachalcone (ISO; 25 μM) combined with rIGF1. The membrane translocation of GFP-AKT was detected after 10 min (**A**), and the intensity of membranous AKT was measured (**B**) Data are means ± SD of quadruplicates (^***^P < 0.001 vs. untreated control; ^##^*P* < 0.01, ^###^*P* < 0.001 vs. DMSO/Ctr; Tukey’s multiple comparisons test). **C** Serum-deprived U2OS cells were treated with ISO (25 μM) with or without recombinant IGF1 (rIGF1, 10 nM) for 6 h, and parallel immunoblots were performed for detecting pAKT, AKT, pmTOR, mTOR, pS6K, S6K, and LC3-II. β-actin (ACTB) was utilized to ensure equal loading (**C**). **D**, **E** U2OS-GFP-LC3 cells transfected with a plasmid coding for AKT^T308D/S473D^ were treated with ISO (25 μM) or torin 1 (300 nM) for 6 h, and GFP-LC3 dots were quantified in (**E**). Scale bar equals 10 μm. Data are means ± SD of quadruplicates (^**^P < 0.01, ^***^P < 0.001 vs. untreated control; ^#^*P* < 0.05, ^##^*P* < 0.01, ^###^*P* < 0.001 vs. WT; Tukey’s multiple comparisons test). **F**, **G** U2OS cells were transfected with a plasmid expressing AKT^T308D/S473D^. Then the cells were serum-deprived and treated with ISO (25 μM) for 6 h. Parallel immunoblot for pAKT, AKT, and LC3-II were performed, and ACTB was used to ensure equal loading. Band intensities of LC3-II and ACTB were assessed, and their ratio (LC3-II/ ACTB) was calculated (**G**). Data are means ± SD of three independent experiments (^***^P < 0.001 vs. untreated control; ^###^*P* < 0.001 vs WT; Tukey’s multiple comparisons test).

ISO is known to inhibit protein kinase B (PKB, best known as AKT)^15,16^. Indeed, U2OS cells stably expressing a GFP-AKT fusion protein responded to stimulation with recombinant insulin growth factor-1 (rIGF1) by a partial translocation of the fluorescent signal to the plasma membrane, reflecting AKT activation. This effect was not detectable for a loss-of-function mutation of AKT consisting of an arginine-to-cysteine mutation in the pleckstrin homology domain of AKT (R25C) (Fig. 2A, B). In addition, ISO inhibited the activating phosphorylation of AKT (Ser473) as well as, downstream of AKT, the phosphorylation of mechanistic target of rapamycin (mTOR) (Ser448), and the mTOR complex 1 (mTORC1)-dependent phosphorylation of S6K (Thr389) (Fig. 2C). Stable transfection of U2OS cells with a constitutively active AKT mutant (T308D/S473D) inhibited the formation of ISO-induced GFP-LC3 puncta (Fig. 2D, E) as well as the lipidation of LC3 (Fig. 2F, G). In conclusion, it appears that ISO stimulates autophagy through the inhibition of AKT.

### ISO induces autophagic flux in vitro and in vivo

**Fig. 3:**
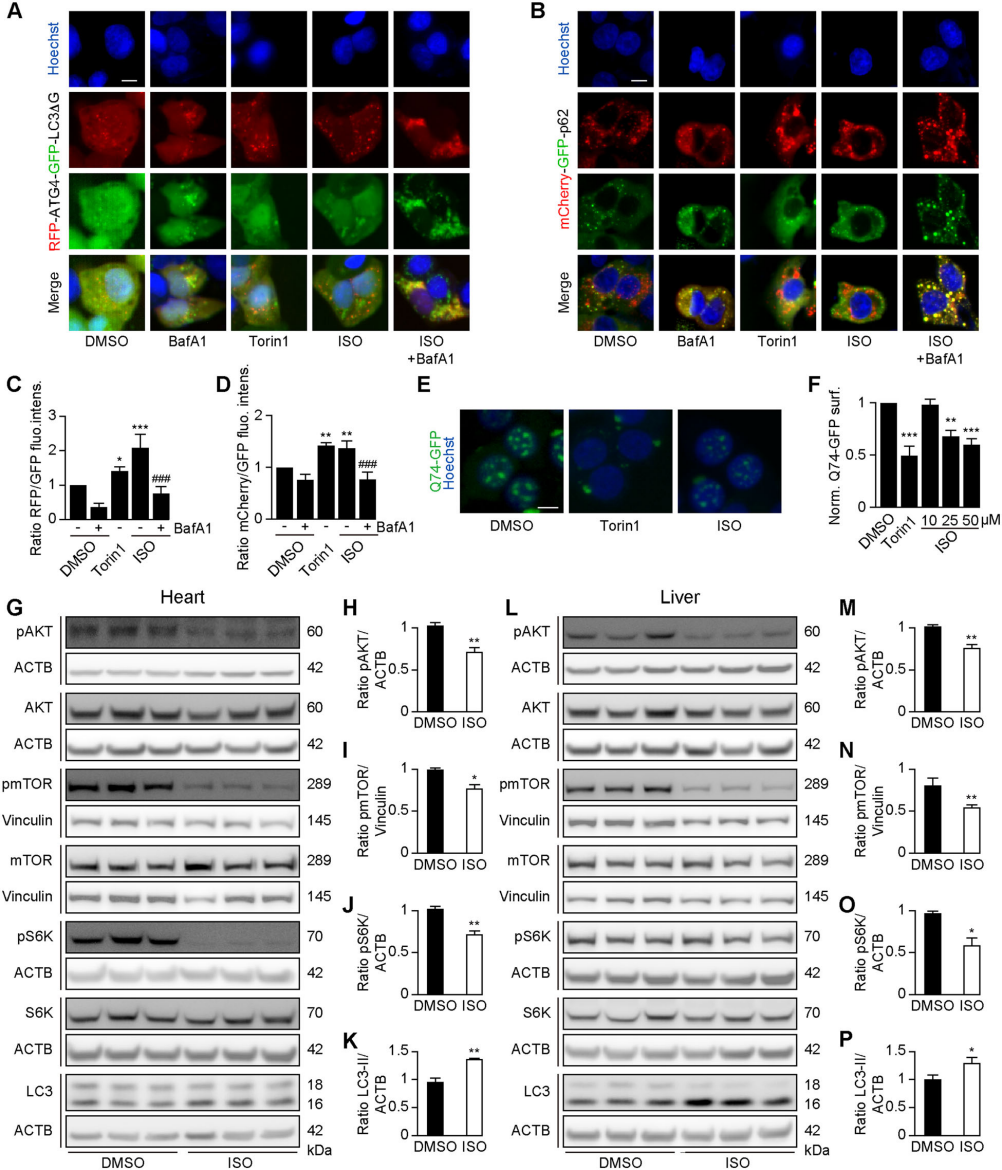
ISO stimulates autophagic flux in vitro and in vivo. **A**–**D** Human osteosarcoma U2OS cells stably expressing the tandem reporter construct GFP-LC3-ATG4-RFP-LC3ΔG (**A**) or the tandem reporter mCherry-GFP-p62 (**B**) were treated with torin 1 (300 nM) or isobacachalcone (ISO; 25 μM) with or without bafilomycin A1 (Baf A1, 100 nM) for 6 h. After fixation, GFP and RFP fluorescence was measured by automated image analysis, and the ratio of RFP to GFP was calculated (**C**, **D**). Scale bar equals 10 μm. Data are means ± SD of quadruplicates (^*^P < 0.05, ^**^P < 0.01, ^***^P < 0.001 vs. untreated control;^###^ *P* < 0.001 vs. without Baf A1; Tukey’s multiple comparisons test). **E**, **F** Rat adrenal gland PC12 cells stably expressing an inducible variant of Q74-GFP were treated with doxycycline (1 μg/mL) for 8 h for the induction of Q74 expression. Then the medium was changed, and ISO (10, 25, 50 μM) was added for 24 h. Torin 1 (300 nM) was used as a positive control. Representative images are shown in (**E**), and GFP-Q74 levels were quantitated in (**F**). Scale bar equals 10 μm. Data are means ± SD of quadruplicates (^**^P < 0.01, ^***^P < 0.001 vs. DMSO/Ctr, Student’s *t* test). **G**–**M** C57BL/6 mice received two *intraperitoneal* (*i.p.*) injections of 20 mg/kg/day ISO (*n* = 3 mice per condition, *n* = 2 experiments). Organs were collected, and representative immunoblots showing regulators and LC3I-to-LC3-II conversion in the heart (**G**–**K**) and in the liver (**L**–**P**). AKT, mTOR, and p70 abundance was evaluated, and parallel samples were probed with phosphoneoepitope-specific antibodies. β-actin (ACTB) or vinculin levels were monitored to ensure equal protein loading (**H**, **J**). Band intensities of pAKT and ACTB, pmTOR and Vinculin, pS6K and ACTB, as well as LC3-II and ACTB, were assessed, and their ratios were calculated (**H**–**K**, **M**–**P**). Data are means ± SD (*n* = 3; (^*^P < 0.05, ^**^P < 0.01 vs. DMSO/Ctr, Student’s *t* test).

Next, we determined whether ISO induces actual autophagic flux by means of several fluorescent reporter-based assays. First, we took advantage of a cell line stably expressing an RFP-ATG4-GFP-LC3ΔG. When expressed in cells, the probe is cleaved into a stable/cytosolic part, RFP-LC3ΔG (that serves as an internal control) and a degradable/quenchable part, GFP-LC3 (which is destroyed by autophagy). Hence, a diminution of the GFP-to-RFP ratio indicates the occurrence of autophagy^17^. ISO consistently induced a decrease in the GFP-to-RFP ratio of cells expressing RFP-ATG4-GFP-LC3ΔG (Fig. 3A, C). We also used cells stably expressing a mCherry-GFP-p62 tandem fusion protein, in which the low pH-sensitive GFP-dependent fluorescence (but less so the pH-resistant mCherry fluorescence) was reduced upon the culture of the cells with ISO (Fig. 1B, D). Similarly, we used a rat adrenal gland (pheochromocytoma) PC12 cell line expressing a tetracycline-inducible variant of Q74-GFP, meaning that the GFP via a polyglutamine tail forms aggregates in the cytoplasm that can be degraded by macroautophagy^18^. Again, we found that ISO reduced the number of Q74-GFP dots in this experimental system, supporting the idea that it indeed stimulates autophagic flux.

Encouraged by these findings, we determined whether ISO might inhibit the AKT pathway and induce autophagy in vivo. Multiple immunoblot experiments indicated that ISO reduces AKT, mTOR, and S6K phosphorylation while it enhances the abundance of LC3-II in the heart or liver of mice receiving intraperitoneal (i.p.) ISO injections. Thus, ISO can stimulate autophagy in vivo. Notably, the in vivo effects of ISO were not accompanied by measurable weight loss, suggesting that ISO is not toxic.

### ISO induces TFEB/TFE3 activation and ER stress

**Fig. 4:**
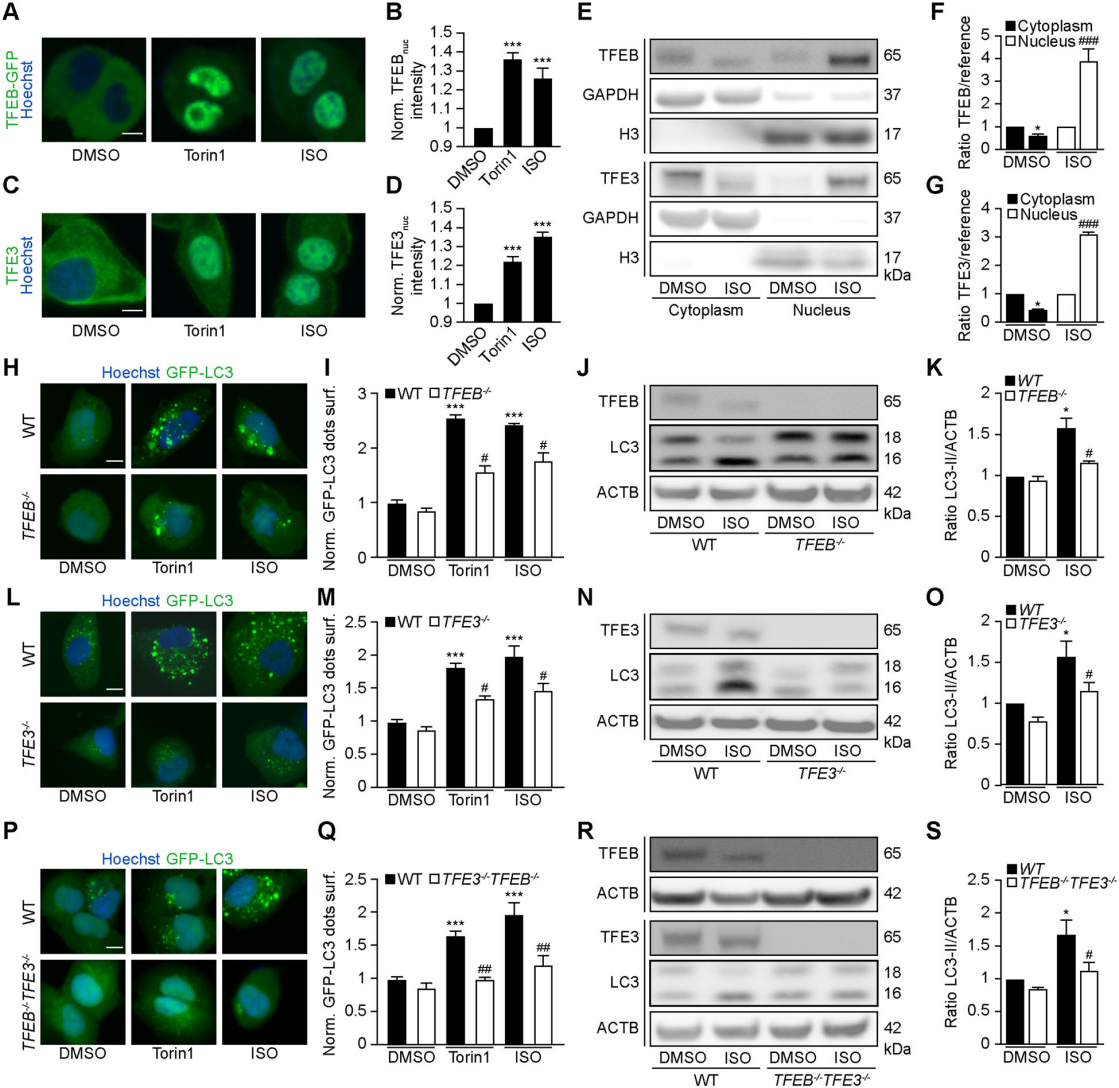
ISO induces TFEB- and TFE3-dependent autophagy. **A**, **B** Human osteosarcoma U2OS cells stably expressing GFP-TFEB fusion protein were treated with torin 1 (300 nM) and isobacachalcone (ISO, 25 μM) for 6 h. Representative images are shown in (**A**) and TFEB translocation was assessed by measuring GFP intensities in the nuclei (**B**). Scale bar equals 10 μm. Data are means ± SD of quadruplicates (^***^P < 0.001 vs. DMSO/Ctr, Student’s *t* test). **C**, **D** U2OS cells were treated with torin 1 (300 nM) and ISO (25 μM) for 6 h, and then, endogenous TFE3 translocation was assessed by immunostaining (**C**). Nuclear TFE3 intensities are depicted in (**D**). Scale bar equals 10 μm. Data are means ± SD of quadruplicates (^***^P < 0.001 vs. DMSO/Ctr, Student’s *t* test). **E**–**G** U2OS cells were treated with ISO (25 μM) for 6 h or were left untreated. Cytoplasmic and nuclear fractions were assessed for nuclear translocation of the transcription factors TFEB and TFE3 in parallel samples by SDS–PAGE. GAPDH and H3 were used to ensure equal loading in the cytoplasmic and nuclear fractions, respectively. Band intensities of TFEB, TFE3, GAPDH, and H3 were assessed and their ratios (TFEB or TFE3/GAPDH, and TFEB or TFE3/H3) were calculated in (**F**, **G**). (^*^P < 0.05, ^**^P < 0.01, ^***^P < 0.001 vs. cytoplasmic DMSO; ^#^*P* < 0.05, ^##^*P* < 0.01, ^###^*P* < 0.001 vs. nuclear DMSO; Tukey’s multiple comparisons test). **H**–**K** U2OS cells stably expressing GFP-LC3 either wild-type (WT) or knockout for TFEB were treated with torin 1 (300 nM) or ISO (25 μM) for 16 h. LC3-II expression and TFEB deficiency were visualized by SDS–PAGE and immunoblot (**J**). Band intensities of LC3-II and β-actin (ACTB) were assessed, and their ratio (LC3-II/ACTB) was calculated in (**K**). Representative images are shown in (**H**), and GFP-LC3 dots were quantified as indicators of autophagy (**I**). Scale bar equals 10 μm. Data are means ± SD of quadruplicates (^***^P < 0.001 vs. untreated control; ^#^*P* < 0.05 vs. WT; Tukey’s multiple comparisons test). **L**–**O** U2OS cells stably expressing GFP-LC3 either WT or knockout for TFE3 were treated with torin 1 (300 nM) and ISO (25 μM) for 16 h. LC3-II expression and TFE3 deficiency were monitored by SDS–PAGE and immunoblot (**N**). Band intensities of LC3-II and ACTB were assessed, and their ratio (LC3-II/ACTB) was calculated in (**O**). Representative images are shown in (**L**), and GFP-LC3 dots were quantified (**M**). Scale bar equals 10 μm. Data are means ± SD of quadruplicates (^*^P < 0.05, *P* < 0.001 vs. untreated control; ^#^*P* < 0.05 vs. WT; Tukey’s multiple comparisons test). **P**–**S** U2OS cell stably expressing GFP-LC3 either wild-type or double knockout for TFEB and TFE3 cells were treated with torin 1 (300 nM) and ISO (25 μM) for 16 h. LC3-II expression and TFEB/TFE3 deficiency were checked in parallel samples by SDS–PAGE and immunoblot (**R**). Band intensities of LC3-II and ACTB were assessed, and the ratio (LC3-II/ACTB) was calculated (**S**). Representative images are shown in (**P**), and GFP-LC3 dots were quantified as indicators of autophagy (**Q**). Scale bar equals 10 μm. Data are means ± SD of quadruplicates (^*^P < 0.05, ^***^P < 0.001 vs. untreated control; ^#^*P* < 0.05, ^##^*P* < 0.01 vs. WT; Tukey’s multiple comparisons test).

U2OS cells exposed to ISO exhibited the translocation of a TFEB-GFP fusion protein from the cytoplasm to the nucleus (Fig. 4A, B). Similarly, TFE3 detectable by immunofluorescence translocated to the nucleus upon culture with ISO (Fig. 4C, D). The nuclear translocation of TFEB and TFE3 could be confirmed by cellular fractionation and immunoblot detection of the two transcription factors in the cytoplasm and nuclei (Fig. 4E–G). Accordingly, knockout of *TFEB* alone (Fig. 4H–K), *TFE3* alone (Fig. 4L–O), or their double knockout (genotype: *TFEB*^−/−^ *TFE3*^−/−^) blunted the induction of autophagic GFP-LC3 puncta and the lipidation of LC3.

**Fig. 5:**
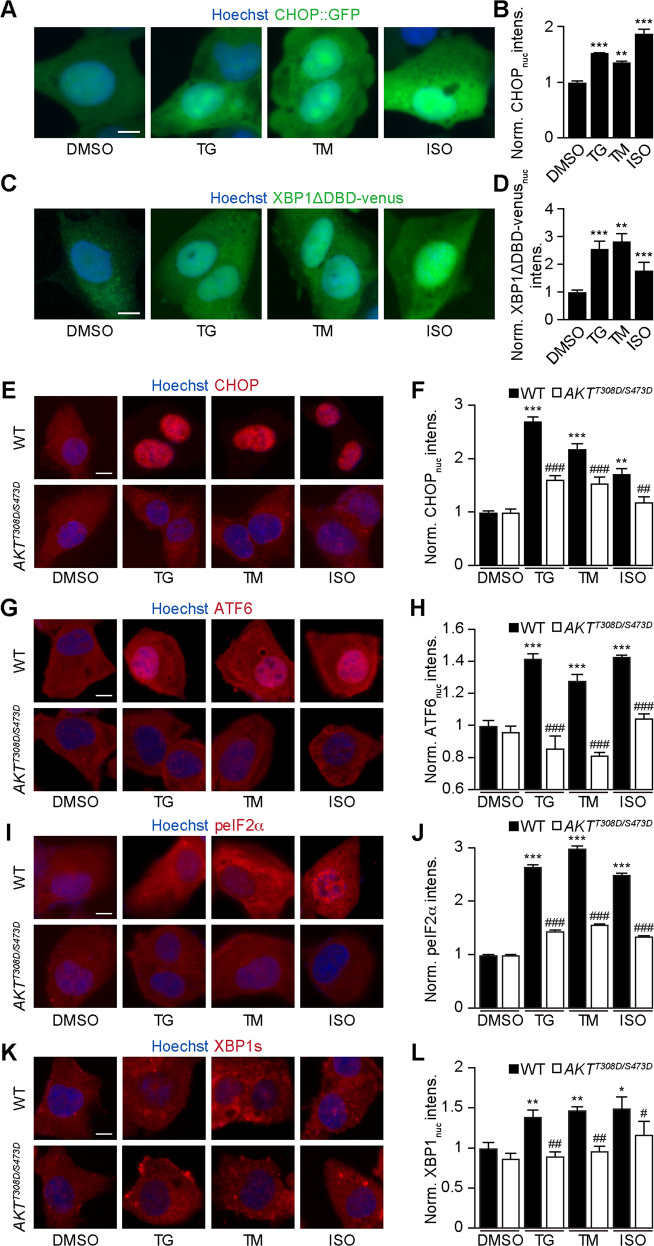
ISO stimulates ER stress via the inhibition of AKT phosphorylation. **A**, **B** Human osteosarcoma U2OS cells stably expressing GFP under the CHOP promoter (CHOP::GFP) were treated with the indicated agents (tunicamycin, TM (3 μM), TG thapsigargin (3 μM), isobacachalcone ISO (25 μM)) for 24 h. GFP nuclear translocation is shown in (**A**), and the average nuclear intensity of GFP was quantified in (**B**). Scale bar equals 10 μm. Data are means ± SD of quadruplicates (^**^P < 0.01, ^***^P < 0.001 vs. untreated control; Student’s *t* test). **C**, **D** U2OS cells stably expressing XBP1ΔDBD-venus (for monitoring venus expression upon alternative splicing of XBP1 mRNA) were treated as indicated for 16 h. XBP1s expression is shown in (**C**), and the average nuclear intensity was measured in (**D**). Scale bar equals 10 μm. Data are means ± SD of quadruplicates (^**^P < 0.01, ^***^P < 0.001 vs. untreated control; Student’s *t* test). **E**–**L** U2OS wild-type (WT) or knock-in for AKT^T308D/S473D^ cells were treated with TM (3 μM), TG (3 μM), ISO (25 μM) 24 h for assessing CHOP, 6 h for measuring peIF2α, and 16 h for monitoring ATF6 and XBP1s. After fixation, the cells were stained with corresponding primary antibodies followed by an AlexaFluor-568 secondary antibody. Nuclei were counterstained with Hoechst 33342. CHOP nuclear expression is shown in (**E**), and the average nuclear intensity of CHOP was quantified in (**F**). ATF6 nuclear translocation is shown in (**G**), and the average nuclear intensity of ATF6 was quantified in (**H**). PeIF2α was assessed by means of immunofluorescence staining (**I**), and the average cytoplasmic intensity of cells was depicted in (**J**). XBP1s activation is shown in (**K**), and the average nuclear intensity was measured in (**L**). Scale bar equals 10 μm. Data are means ± SD of quadruplicates (^*^P < 0.05, ^**^P < 0.01, ^***^P < 0.001 vs. untreated control; ^#^*P* < 0.05, ^##^*P* < 0.01, ^###^*P* < 0.001 vs. WT; Tukey’s multiple comparisons test).

In U2OS cells equipped with biosensors of endoplasmic reticulum (ER) stress, we found that ISO induced the upregulation of CHOP (measured by using a GFP gene inserted into the genome under the control of the CHOP promoter, Fig. 5A, B) and activated the IRE1/XBP1 axis (measured by means of an XBP1ΔDBD-venus fusion protein^19^ that is only in-frame for venus, a variant of GFP, when XBP1 has been spliced by IRE1, Fig. 5C, D). Similar results were obtained when signs of ER stress were measured by immunofluorescence to detect the nuclear presence of CHOP (Fig. 5E, F) and ATF6 (Fig. 5G, H), the phosphorylation of eukaryotic initiation factor 2α (eIF2α) on serine 51 (Fig. 5I, J) and the expression of the spliced isoform of XBP1 (XBP1s) (Fig. 5K, L). In most of the cases, the ISO-induced signs of ER stress were comparable in magnitude to those induced by the positive controls thapsigargin (TG) and tunicamycin (TM) (Fig. 5A–L). Moreover, the expression of constitutively active AKT mutant blunted the signs of ER stress induced by ISO (Fig. 5E–L).

Interestingly, a crosstalk between the pro-autophagic and the ER stress-inducing activities of ISO was observed. Thus, *TFEB*^−/−^ *TFE3*^−/−^ cells exhibited a reduced activation of CHOP (Supplementary Fig. S1A, B) and ATF4 (Supplementary Fig. S1C, D). Such a reduced CHOP and ATF4 activation was also found for the single-gene knockout of *TFEB* or *TFE3* (Supplementary Fig. S2). Cells lacking the eIF2α kinase 3 (EIF2AK3, best known as PERK) exhibited reduced phosphorylation of eIF2α in response to ISO (Supplementary Fig. S1E, F), coupled to reduced formation of autophagic RFP-LC3 puncta (Supplementary Fig. S1G, H). Both the knockout of PERK and a knock-in mutation of eIF2α rendering it non-phosphorylable (due to the substitution of serine 51 by an alanine residue: S51A) significantly reduced the activation of TFE3 by ISO (Supplementary Fig. S1I–L). These findings suggest molecular crosstalk between the TFEB/TFE3 and the PERK/eIF2α pathways triggered by ISO.

### ISO improves the outcome of immunogenic chemotherapy

**Fig. 6:**
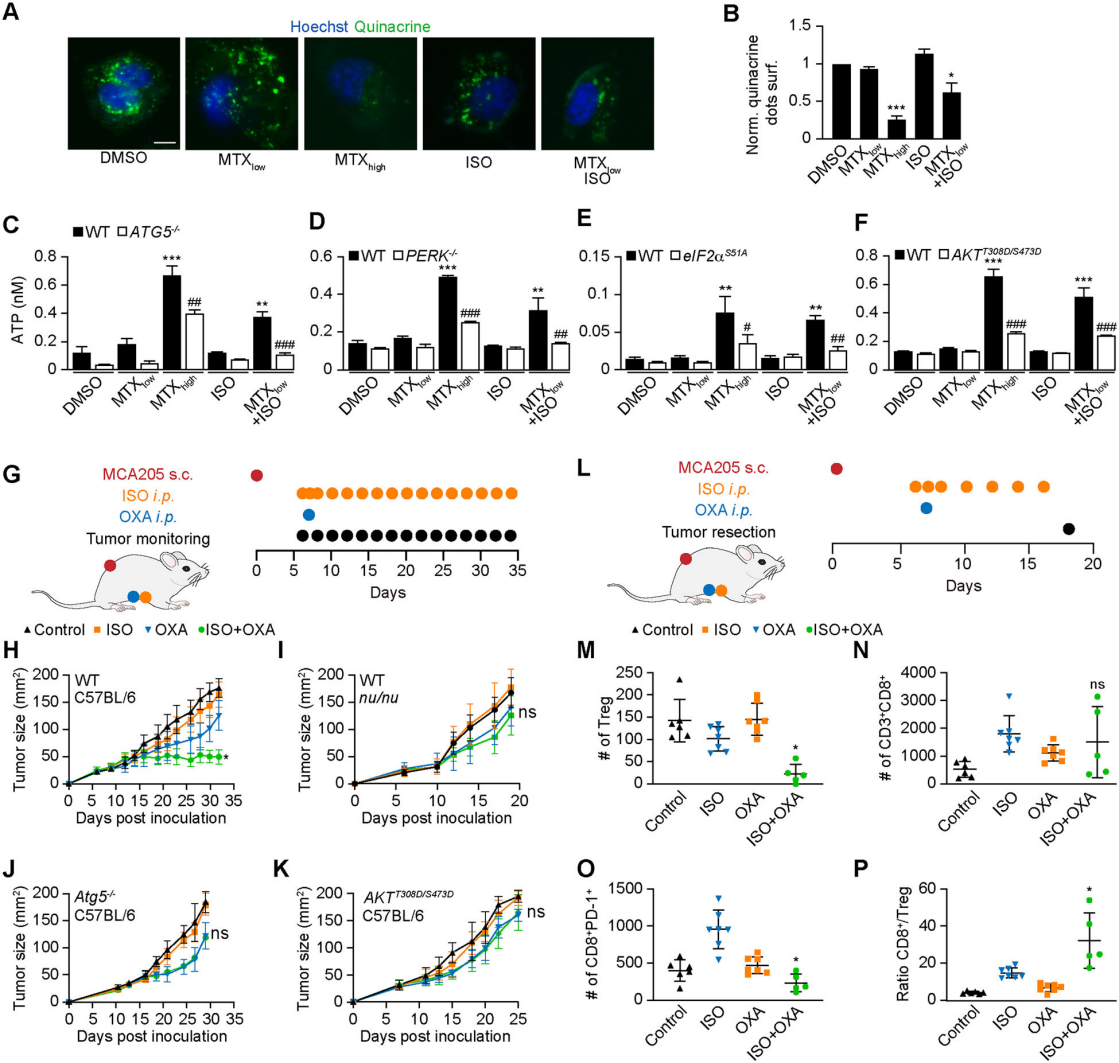
ISO mediates improvement of anticancer chemotherapy. **A**, B Human osteosarcoma U2OS cells were treated with isobacachalcone (ISO, 25 μM) in the presence of low doses of the immunogenic cell death (ICD) inducer mitoxantrone (MTX, 1 μM) for 6 h. High-dose MTX (5 μM) was used as a positive control. The ATP-sensitive agent quinacrine was used to monitor intracellular ATP content. Representative images are shown in (**A**), and the abundance of quinacrine was quantified in (**B**). Scale bar equals 10 μm. Data are means ± SD of quadruplicates (^*^P < 0.05, ^***^P < 0.001 vs. untreated control Tukey’s multiple comparisons test). **C**–**F** Human neuroglioma H4 cell stably expressing GFP-LC3 either wild-type (WT) or ATG5 knockout, human osteosarcoma U2OS wild-type, PERK knockout or eIF2α^S51A^ knock-in cells, murine fibrosarcoma MCA205 WT or AKT^T308D/S473D^ knock-in cells were treated with ISO (25 μM) alone or in combination with low doses of MTX (1 μM) for 6 h, as described above. High-dose MTX (5 μM) was used as a positive control. Extracellular ATP levels were measured by luciferase conversion (^**^P < 0.01, ^***^P < 0.001 vs. untreated control; ^#^*P* < 0.05, ^##^*P* < 0.01, ^###^*P* < 0.001 vs. WT; Tukey’s multiple comparisons test). **G**, **L** Schematic overview of the in vivo treatment of murine fibrosarcoma MCA205-bearing mice with oxaliplatin (OXA) and ISO, alone or in combination. **H**–**K** Growth kinetic of murine fibrosarcoma MCA205 cells WT (**H**), Atg5KD (**J**), or AKT^T308D/S473D^ knock-in (**K**) growing in immunocompetent C57BL/6 mice, treated as indicated in (**G**). Athymic mice (nu/nu) mice (**I**) were inoculated subcutaneously with murine fibrosarcoma MCA205 cells. When tumors became palpable, mice received a systemic intraperitoneal injection of ISO alone or together with OXA. *n* ≥ 6 mice per group. Results (means ± SD tumor growth curves) are plotted (^*^P < 0.05 or ns, not statistically significant vs. OXA). **M**–**P** Murine fibrosarcoma MCA205 cells were evolving in immunocompetent C57BL/6 mice, treated as indicated in (**L**). Cytofluorometric characterization of tumor-infiltrating lymphocytes, in particular CD4^+^FOXP3^+^CD25^+^ regulatory T cells (Treg) (**M**), CD3^+^CD8^+^ cytotoxic T lymphocytes (**N**), CD8^+^PD1^+^ T lymphocytes (**O**), and the ratio of CD3^+^CD8^+^ T cells over Treg (**P**) are depicted. Data are means ± SD (*n* ≥ 5) (^*^P < 0.05 or ns, not statistically significant vs. OXA; Student’s *t* test).

Although ISO alone had rather scarce cytotoxic activities, it was able to amplify the ATP release induced by treatment of U2OS cells with low doses of an ICD inducer (mitoxantrone, MTX), as determined by staining of cells with the ATP biosensor quinacrine (Fig. 6A, B) or by measuring ATP in the supernatant of the cells using a biochemical assay (Fig. 6C–F). ATP is released from stressed cancer cells in an autophagy-dependent fashion^20,21^ and acts in the extracellular space as an important chemotactic factor that attracts myeloid immune effectors into the tumor bed, thereby setting off the molecular cascade that permits anticancer immune responses in the context of ICD^22,23^. In contrast, ISO did not affect other autophagy-independent hallmarks of ICD^24^, including surface exposure of calreticulin or the release of high mobility group protein B1 from low-dose MTX-treated cells (Supplementary Fig. S3). Of note, the knockouts of ATG5 (Fig. 6C) or PERK (Fig. 6D), the S51A mutation of eIF2α (Fig. 6E) or the expression of a constitutively active AKT mutant (Fig. 6F) reduced the ATP release induced by the combination of low-dose MTX and ISO, supporting the idea that the aforementioned pathways are important for this phenomenon.

Next, we determined the capacity of ISO to enhance the efficacy of ICD-inducing chemotherapy in vivo, using immunocompetent mice-bearing syngeneic cutaneous MCA205 fibrosarcomas. We chose this type of methylcholantrene-induced tumor because it is well characterized in immunosurveillance models^25,26^, and because its growth under the skin can be considered as orthotopic. Once MCA205 tumors had been established, the mice received the ICD inducer oxaliplatin (OXA), ISO or the combination (OXA + ISO) while negative controls received vehicle alone (Fig. 6G). Of note, the combination (OXA + ISO) allowed for tumor growth control in conditions in which ISO and OXA alone had no or little effect, respectively (Fig. 6H). The anticancer activity depended on the immune system since it was lost in mice lacking mature T cells due to the *nu*/*nu* mutation that causes athymia (Fig. 6I). Moreover, tumor cells engineered to lack *Atg5* or to express constitutively active AKT failed to respond to the ISO/OXA combination treatment in the immunocompetent setting (Fig. 6J, K). Analysis of the immune infiltrates of the tumors treated with ISO, OXA, or ISO + OXA (Fig. 6L) revealed that the combination treatment was particularly efficient in reducing regulatory T cells (Tregs, defined as CD3^+^CD4^+^FoxP3^+^ cells), in improving the ratio of CD8^+^ cytotoxic T lymphocytes (CTLs) over Tregs and in reducing the expression of the exhaustion marker PD-1 on CTLs (Fig. 6M–P). In conclusion, ISO stimulates anticancer immunity in the context of ICD-inducing chemotherapy.

## Discussion

Here, we identified ISO as an autophagy inducer that inhibits AKT and mTORC1 activity and activates the pro-autophagic transcription factors TFEB and TFE3, which both are known to be activated by mTORC1 inhibition^27,28^. We also found that ISO activates a broad ER stress response including the PERK-dependent phosphorylation of eIF2α, as a sign of the integrated stress response, which is known to be required for autophagy induction^29–31^ as well as for the induction of ICD^32–37^. The two pathways, autophagy and ER stress induced by ISO exhibited crosstalk in thus far that (i) they both are inhibited by constitutively active AKT, (ii) TFEB/TFE3 knockout does not only reduce autophagy but also signs of ER stress, and (iii) PERK knockout or substitution of eIF2α by a non-phosphorylable mutant reduces TFEB/TFE3 activation and autophagy. Beyond these in vitro phenomena, ISO induced autophagy in vivo, in mouse tissues, and enhanced the immune response induced by immunogenic chemotherapy against established tumors, thus improving tumor growth control through mechanisms that rely on T cells as well as AKT inhibition and autophagy induction in the cancer cells.

ISO is a chalcone that was first isolated from the multipurpose medical plant *Psoralea corylifolia*. Reportedly, ISO possesses a wide spectrum of antibacterial^38,39^ antifungal^40^ antiparasitic^41^, antiviral^42,43^, antitubercular^44^, antithrombotic^45,46^, antiinflammatory^47,48^, antioxidant^49^, antiobesity^50^, and phytoestrogene^51^ activities. Hence, ISO has a very broad range of biological activities. In cell-free enzymatic assays, ISO inhibits beta-secretase^52^, acyl-coenzyme A: cholesterol acyltransferase^53^, severe acute respiratory syndrome coronavirus (SARS-CoV) papain-like protease^54^, protein tyrosine phosphatase 1B (PTP1B)^55^, carboxylesterase 2^56^, and pancreatic lipase^57^, suggesting that ISO can act on multiple pharmacological targets, shedding doubts on its specificity. Based on its broad effects, it might be suspected that ISO has direct immunostimulatory effects that help to improve immunosurveillance in the context of ICD-inducing chemotherapies. Indeed, autophagy induction may stimulate dendritic and T-cell functions^23,58,59^.

In vitro, ISO reduces Aβ42 aggregation in SH-SY5Y cells^60^ and the tumor necrosis factor-α (TNFα)-induced atrophy of C2C12 myotubes^61^. In rodents, ISO attenuates Parkinson’s disease induced by the toxin 1-methyl-4-phenyl-1,2,3,6- tetrahydropyridine (MPTP)^62^, sephadex-induced lung injury^63^, as well as streptozotocin-induced diabetic nephropathy^64^. This suggests that ISO has a wide range of cytoprotective effects that might be explained by its autophagy-inducing activity.

With respect to its anticancer effects, ISO reportedly suppresses skin tumor promotion in an in vivo two-stage mouse skin carcinogenesis test using 7,12-dimethylbenz[a]anthracene (DMBA) as an initiator and 12-O-Tetradecanoylphorbol-13-acetate (TPA) as a promoter^65^. ISO has cytotoxic effects on neuroblastoma^66^, multiple myeloma cells^67,68^, leukemia^69^, as well as on chemoresistant carcinoma and glioblastoma cell lines^70^, enhances TRAIL-induced apoptosis in prostate cancer and cervical carcinoma cells^71^, and reduces melanin production by B16 melanoma cells^72^. Here, we found that ISO failed to inhibit the growth of fibrosarcomas in mice when used as a standalone treatment, yet ameliorated the efficacy of ICD-inducing chemotherapy through an improved anticancer immune response. The absence of antitumor efficacy of ISO, when used as a standalone treatment, may be linked to suboptimal dosing as well as to its pharmacokinetics, knowing that ISO has a half-life of ~6 h in rats^73^. However, we have observed as a general pattern that autophagy induction with non-toxic agents is not sufficient to inhibit tumor growth of established tumors in mice. Thus, the biological activity of ISO is reminiscent of other autophagy inducers including 3,4-DMC^13^, hydroxycitrate, resveratrol, spermidine^74,75^, and thiostreptone^76^, all of which can ameliorate the therapeutic activity of ICD inducers in suitable mouse models but lack intrinsic anticancer properties.

Although ISO has multiple pharmacological effects and targets, several of the in vitro effects of ISO correlated with the inhibition of the AKT/mTORC1 pathway, and expression of a constitutively active AKT mutant largely reversed the ISO-induced signs of cellular stress including autophagy (with its upstream events, mTORC1 inhibition and TFEB/TFE3 activation) and ER stress (at all levels of the unfolded stress response, including its PERK/eIF2α/ATG4/CHOP, ATG6, and IRE1α/XBP1 arms), as shown in human U2OS cells. Moreover, mouse cancer cells stably expressing a constitutively active AKT enzyme (or lacking the essential autophagy gene *Atg5*) became resistant against the anticancer activity of ISO combined with ICD induction, suggesting some sort of ‘specificity’ for the ISO effect. However, at this point, it is not clear whether ISO may directly inhibit AKT or an enzyme upstream of AKT (such as phosphatidylinositol 3-kinases). Reportedly, ISO inhibits PTP1B^55^, which would result in the activation, not the inhibition of the AKT pathway. Hence, the precise molecular target of ISO remains elusive.

ISO was initially isolated from *Psoralea corylifolia*, but has also been identified in other plants, including in *Angelica keiskei*^50^, *Artocarpus species*^46^*, Cullen corylifolium*^77^*, Dorstena barteri*^38^, *Erythrena fusca*^78^, *Fatoua pilosa*^44^, *Morus alba*^79^, and *Piper longum*^72^. This suggests that ISO is rather prevalent in plants, perhaps contributing to the broad pro-health effects of plant-enriched diets^80,81^. However, additional studies are required to confirm this conjecture.

In summary, here we identified a particular chalcone, ISO, as a potent autophagy inducer that acts in vitro and in vivo, on human cell lines and mouse organs, respectively. Through the induction of autophagy, ISO is able to stimulate anticancer immune responses in the context of immunogenic chemotherapy.

## Materials and methods

### Cell culture and chemicals

Culture media and supplements for cell culture were obtained from Life Technologies (Carlsbad, California, USA) and plastic materials came from Greiner Bio-One (Kremsmünster, Austria) and Corning (Corning, NY, USA). Rat adrenal gland PC12 cells stably expressing doxycycline-inducible Q74-GFP were cultured in Roswell Park Memorial Institute (RPMI)-1640 containing 5% fetal bovine serum and 10% horse serum^82^. Human neuroglioma H4 cells, human osteosarcoma U2OS cells, MCA205 murine fibrosarcoma, and all the other cells were maintained in Dulbecco’s modified Eagle’s medium (DMEM), supplemented with 10% (v/v) fetal bovine serum (FBS), 10 U mL^−1^ penicillin sodium and 10 μg mL^−1^ streptomycin sulfate at 37 °C in a humidified atmosphere with 5% CO_2_. TFEB-deficient (*TFEB*^−*/*−^), TFE3-deficient (*TFE3*^−*/*−^), TFEB and TFE3-double deficient (*TFEB*^−*/*−^*TFE3*^−*/*−^), ATG5-deficient (*ATG5*^−*/*−^), and PERK-deficient (*PERK*^−*/*−^) U2OS-GFP-LC3 cell lines and TFEB and TFE3-double deficient (*TFEB*^−*/*−^*TFE3*^−*/*−^) in H4-GFP-LC3 cells were generated by means of the CRISPR/Cas-mediated genome editing, as per the manufacturer’s recommendations^13^. U2OS cells stably expressing RFP-LC3 bearing a mutant non-phosphorylation of eIF2α (eIF2α^S51A^) were constructed using the CRISPR-Cas9 technology as previously detailed^31^. In addition, U2OS cells stably expressing GFP-TFEB, CHOP::GFP, and XBP1s-DDBD-venus were generated by our group in the past^13,36^. MCA205 cells stably expressing shRNAs interfering with the expression of TFE3/TFEB or ATG5, and a mutant phosphorylation AKT T308D/S473D were also constructed as recommended by the manufacturer^13,74,83^. The Polyphenolic Natural Compound Library library and ISO were purchased from TargetMol (Boston, Massachusetts, USA); torin 1 (TOR), thapsigargin (TG), tunicamycin (TM), bafilomycin A1 (Baf A1), mitoxantrone (MTX), and oxaliplatin (OXA) were obtained from Sigma-Aldrich (St. Louis, Missouri, USA).

### High-content microscopy

Human osteosarcoma U2OS and neuroglioma H4 cells stably expressing GFP-LC3 or RFP-LC3 and rat adrenal gland PC12 cells stably expressing doxycycline-inducible Q74-GFP were seeded in 384-well black imaging plates at a density of 2000 cells per well and allowed to adapt for overnight. Cells were treated with the indicated agents for 6 h, subsequently, cells were fixed with 3.7% paraformaldehyde (PFA, w/v in PBS) (F8775, Sigma-Aldrich) at 4 °C overnight and stained with 1 µg/ml Hoechst 33342 in PBS. Moreover, 2000 U2OS cells either wild-type or stably expressing HMGB1-GFP/CALR-RFP, GFP-ATF6, CHOP::GFP, GFP-TFEB, or XBP1-DDBD-venus were seeded in 384-well black imaging plates (Greiner Bio-One) and let adhere overnight. Cells were then treated for 6 h to detect TFEB translocation, 16 h to assess ATF6 translocation and spliced XBP1 (XBP1s) levels, or 24 h to measure CHOP promoter activity. For CALR redistribution and HMGB1 release, cells were incubated for 8 h or 24 h respectively. Next, cells were fixed with 3.7% formaldehyde supplemented with 1 μg/ml Hoechst 33342 (H3570, Thermo Fisher Scientific) at 4 °C overnight. Subsequently, the fixative was exchanged to PBS, and the plates were analyzed by automated microscopy. Image acquisition was performed using an ImageXpress Micro XL automated microscope (Molecular Devices, Sunnyvale, CA, USA) equipped with a ×20 PlanApo objective (Nikon, Tokyo, Japan), followed by automated image processing with the custom module editor within the MetaXpress software (Molecular Devices). At least four view fields were acquired per well, and experiments involved at least triplicate assessment. Cellular regions of interest, cytoplasm and nucleus, were defined and segmented by using the MetaXpress software (Molecular Devices). After exclusion of cellular debris and dead cells from the dataset, parameters of interest were normalized, statistically evaluated, and graphically depicted with R software. Using R, images were extracted and pixel intensities scaled to be visible (in the same extent for all images of a given experiment).

### Immunofluorescence

Human osteosarcoma U2OS cells were treated for 6 h to detect eIF2α phosphorylation (PeIF2α) and TFE3, 16 h to assess ATF6 and spliced XBP1 (XBP1s) levels, or 24 h to measure CHOP expression. Then cells were fixed by 3.7% PFA at 4 °C overnight. For staining, fixed cells were then permeabilized with 0.1% Triton X100 on ice, and blocked with 5% bovine serum albumin (BSA, w/v in PBS) for 1 h. Next, cells were incubated with antibodies specific to TFE3 (#ab93808, 1:400, Abcam), phospho-eIF2 alpha (Ser51) (#ab32157, 1:1000, Abcam), ATF6 (#ab37149, 1:200, Abcam), XBP1 (#ab37152, 1:250, Abcam) or CHOP (#2895, 1:500, Cell Signaling Technology) at 4 °C overnight. After washed by PBS twice, AlexaFluor conjugates (Thermo Fisher Scientific) against the primary antibody were applied for 2 h at RT. Finally, cells were washed and imaged by automated fluorescence microscopy as described above. The nuclear intensity of TFE3, ATF6, XBP1s or CHOP and cytoplasmic intensity of phospho-eIF2α (Ser51) were measured and normalized on Ctrl.

### Immunoblotting

The tissues (~30 mg) were dissociated in Precellys lysing tubes (#CK28_2 mL, Bertin Technologies SAS, Montigny-le-Bretonneux, France) containing 1 mL of radioimmunoprecipitation assay buffer (RIPA) lysis buffer (#89901, Invitrogen, Carlsbad, CA, USA) by using the Precellys 24 homogenizer (Bertin Technologies SAS) at 6500 rpm for 5 min, followed by spinning at 14,000×*g* for 15 min to collect the supernatant that contains soluble proteins. For cells, the protein extracts were dissolved in RIPA buffer and obtained by ultrasonication for 3 × 10 s and centrifuging at 12,000×*g* for 15 min to collect the supernatant that contains soluble proteins. Protein concentration was measured by means of the BCA Assay (Bio-Rad, Hercules, CA, USA). The protein solution was mixed with 4× loading buffer (# NP0008, Invitrogen), and denatured at 100 °C for 15 min before subjected to western blotting. The total protein (~30 μg) were resolved on 4–12% NuPAGE Bis-Tris protein gels (#NP0322, Thermo Fisher Scientific) and electrotransferred to 0.2 μM polyvinylidene fluoride (PVDF) membranes (#1620177, Bio-Rad). The membranes were blocked with 0.05% Tween 20 (#P9416, Sigma-Aldrich) v-v in Tris-buffered saline (TBS) (TBST) (#ET220, Euromedex) supplemented with 5% nonfat powdered milk (w:v in TBS), followed by an overnight incubation at 4 °C with primary antibodies specific for LC3B (#2775, 1:1000, Cell Signaling Technology), HA (#ROAHAHA, 1:1000, Sigma-Aldrich), phospho-P70 (Thr389) (#9234, 1:1000, Cell Signaling Technology), P70 (#9202, 1:1000, Cell Signaling Technology), TFEB (#4240, 1:1000, Cell Signaling Technology), TFE3 (#ab93808, 1:1000, Abcam), phospho-AKT (Ser473) (#4060, Cell Signaling Technology), AKT (#4691, Cell Signaling Technology), phospho-mTOR (Ser2448) (#2971, Cell Signaling Technology), mTOR (#2983, Cell Signaling Technology), H3 (#9715, 1:1000, Cell Signaling Technology). Membranes were washed three times with TBST for 10 min each before incubated with HRP-conjugated goat-anti-rabbit secondary antibody (CliniScience) for 2 h at room temperature. At last, the membranes were washed again and subjected to chemiluminescence detection with the Amersham ECL Prime detection reagent kit (GE Healthcare, Piscataway, NJ, USA) on an ImageQuant LAS 4000 software-assisted imager. Samples from cells or organs were aliquoted and run on separate gels. Equal loading was controlled by Coomassie staining. The abundance of control proteins (such as β-actin (ACTB, #ab 20727, 1:10000, Abcam), glyceraldehyde-3-phosphate dehydrogenase (GAPDH) (#2118, 1:5000, Cell Signaling Technology), vinculin (#13901, 1:1000, Cell Signaling Technology) vinculin, or non-phosphorylated proteins such as AKT, S6K, mTOR) in each sample was determined in parallel samples. Quantification was performed by densitometry using the Image J software.

### Nuclear extraction experiment

U2OS-GFP-LC3 cells were collected and processed with the Nuclear Extraction Kit (#ab113474, Abcam) following the manufacturer’s methods. The GAPDH antibody (#2118, 1:1000, Cell Signaling Technology) was used as the cytoplasmic control, and H3 (#9715, 1:1000, Cell Signaling Technology) was selected as the nuclear control.

### Detection of protein deacetylation

U2OS cells stably expressing GFP-LC3 (~2000 cells/well) were seeded in 384-well microplates overnight. After experimental treatments, cells were fixed with 3.7% PFA containing 10 μg/ml Hoechst 33342 overnight at 4 °C. Thereafter, cells were incubated with an antibody specific for acetyl-alpha-tubulin (#5335, 1:500, Cell Signaling Technology) in 5% BSA (w/v in PBS) for 1 h to block non-specific binding sites and acetylated tubulins, followed by overnight incubation at 4 °C with an antibody specific to acetylated lysine residues (#623402, 1:400, BioLegend, San Diego, California, USA). After washing three times with PBS, cells were incubated in AlexaFluor-568-conjugated secondary antibodies (Life Technologies) for 2 h at room temperature. Fluorescent images were acquired and analyzed as described before.

### ATP release assays

Intracellular ATP levels were detected by quinacrine stain assay (Calbiochem) kits, subsequently, the images of quinacrine were obtained by high-content microscopy and the cytoplasmic intensity of quinacrine was quantitated described above. Extracellular ATP levels were measured by the ENLITEN ATP Assay System Bioluminescence Detection Kit (Promega, Madison, Michigan, USA; #FF2000) following the manufacturer’s methods. Luminescence was detected by means of a Paradigm I3 multimode plate reader (Molecular Devices).

### Animal experimentation

The animal experiments were approved by the Gustave Roussy ethical committee with project number 24771–2020032413235413, and all procedures were performed under the governmental and institutional guidelines and regulations. All mice were maintained in a temperature-controlled and pathogen-free environment with 12-h light/dark cycles, with food and water ad libitum. Animal experiments were conducted in compliance with the EU Directive 63/2010 and protocols 2019_030_20590 and were approved by the Ethical Committee of the Gustave Roussy Campus Cancer (CEEA IRCIV/IGR no. 26, registered at the French Ministry of Research).

For tumor growth experiments, 7-week-old female wild-type C57BL/6 mice or athymic female nude mice (*nu/nu*) were obtained from Envigo, France (Envigo, Huntingdon, UK). MCA205 wild-type (WT), or continuous activation of AKT T308D/S473D cells (4 × 105), MCA205 cells carrying an ATG5 knockdown (WT, 6 × 105) were subcutaneously injected into C57BL/6 hosts. When tumors became palpable, mice were treated with 20 mg/kg ISO dissolved in corn oil (Sigma-Aldrich) or an equivalent volume of vehicle alone or in combination with 10 mg/kg oxaliplatin (OXA, Sigma-Aldrich) by intraperitoneal injection. On the following days, mice well-being and tumor growth were monitored and documented. Animals were sacrificed when tumor size reached the ethical endpoint or signs of obvious discomfort were observed following the EU Directive 63/2010 and our Ethical Committee advice.

### Ex vivo phenotyping of the tumor immune infiltrate

Tumors were harvested, weighed, and transferred on ice into gentleMACS C tubes (Miltenyi Biotec, Bergisch Gladbach, Germany) containing 1 mL of RPMI medium. Tumors were dissociated first mechanically with scissors, then enzymatically using Miltenyi Biotec mouse tumor dissociation kit (Miltenyi Biotec) and a GentleMACS Octo Dissociator according to the manufacturer’s instructions. The dissociated bulk tumor cell suspension was resuspended in RPMI-1640, sequentially passed through 70-μm MACS Smart-Strainer (Miltenyi Biotec), and washed twice with PBS. Finally, bulk tumor cells were homogenized in PBS at a concentration corresponding to 250 mg of the initial tumor weight per milliliter. Prior to staining of tumor-infiltrating lymphocytes (TILs) for flow cytometry analysis, samples (~50 mg) were incubated with LIVE/DEAD^®^ Fixable Yellow Dead Cell dye (Thermo Fisher Scientific) to discriminate viable cells from damaged cells. Fc receptors were blocked with anti-mouse CD16/CD32 (clone 2.4G2, Mouse BD Fc Block, BD Pharmingen) before staining with fluorescent-labeled antibodies targeting T-cell surface markers. Surface staining of murine immune cell populations infiltrating the tumor was performed with the following fluorochrome-conjugated antibodies: anti-CD45-AF700, anti-CD3-BV421, anti-CD8-PE, anti-CD4-Percp.Cy5.5, anti-CD25-PE/Cy7, and anti-PD-1-APC/Cy7 (BioLegend). Then, cells were fixed and permeabilized in eBioscience Foxp3/Transcription Factor Staining Buffer (Thermo Fisher Scientific) and stained for intracellular Foxp3. Finally, stained samples were run through a BD LSR II flow cytometer. Data were acquired using BD FACSDiva software (BD Biosciences) and analyzed using FlowJo software (TreeStar). Absolute counts of leukocytes and tumor cells were normalized considering the following parameters: the weight of the harvested tumor and total volume of the dissociated tumor cell suspension (cell concentration typically set to 250 mg/mL in PBS), the proportion of the whole-cell suspension, and proportion of the cell suspension used for cytometry.

### Statistical analysis

Unless otherwise mentioned, data are reported as means ± SD of triplicate determinations, and experiments were repeated at least three times yielding similar results. Statistical significance was assessed by Student’s *t* test. TumGrowth and GraphPad were used to analyze in vivo data arising from murine models^84^. TumGrowth is available at Github/Kroemerlab. *P* values of 0.05 or less were considered to denote significance (^*^P < 0.05; ^**^P < 0.01; ^***^P < 0.001; ns, not significant).

## Supplementary information

Supplemental figure legends

Figure S1

Figure S2

Figure S3
