# Supplementary material for: Isobacachalcone induces autophagy and improves the outcome of immunogenic chemotherapy
Source: Cell Death Dis. 2020 Nov 26;11(11):1015. doi: 10.1038/s41419-020-03226-x (PMC7690654; doi:10.1038/s41419-020-03226-x)
Supplement: Supplementary file 1 — Supplemental figure legends [file 41419_2020_3226_MOESM1_ESM.docx]

## Supplemental figures legends

**Figure S1. ISO depends on the crosstalk between the UPR and autophagy**

(A-D) Human osteosarcoma U2OS wild type (WT) or double knockout for TFEB and TFE3 were treated with tunicamycin (TM, 3 μM), thapsigargin (TG, 3 μM), isobacachalcone (ISO, 25 μM) for 24 h. After fixation, the cells were stained with CHOP-specific and ATF4-specific antibodies followed by an AlexaFluor-568 secondary antibody. Nuclei were counterstained with Hoechst 33342. The nuclear expressions of CHOP (A) and ATF4 (C) were shown, and the average nuclear intensity of CHOP (B) and ATF4 (D) were quantified. Scale bar equals 10 μm. Data are means ± SD of quadruplicates (^*^*p* < 0.05, ^**^*p* < 0.01, ^***^*p* < 0.001 vs. untreated control; ^#^*p* < 0.05, ^##^*p* < 0.01, ^###^*p* < 0.001 vs. WT; Tukey’s multiple comparisons test).

(E-F) U2OS cells WT or knockout for PERK were treated with TM (3 μM), TG (3 μM) or ISO (25 μM) for 6 h. After fixation, phosphorylation of eIF2α (peIF2α) was assessed by means of immunofluorescence staining (E) and the average cytoplasmic fluorescence intensity is depicted in (F). Scale bar equals 10 μm. Data are means ± SD of quadruplicates (^**^*p* < 0.01, ^***^*p* < 0.001 vs. untreated control;^###^*p* < 0.001 vs. WT; Tukey’s multiple comparisons test).

(G-H) U2OS stably expressing RFP-LC3 either WT or knock-in for eIF2α^S51A^ were treated with torin 1 (300 nM) or ISO (25 μM) for 6 h. After fixation, RFP-LC3 was measured and normalized as percent of Ctrl in (H). Representative images of RFP-LC3 dots were shown in (G). Scale bar equals 10 μm. Data are means ± SD of quadruplicates (^***^*p* < 0.001 vs. untreated control; ^###^*p* < 0.001 vs. WT; Tukey’s multiple comparisons test).

(I-J) U2OS cells wild-type or knockout for PERK were treated with torin 1 (300 nM) or ISO (25 μM) for 16 h. After fixation, TFE3 was assessed by means of immunofluorescence staining (I) and the average nuclear fluorescence intensity is depicted (J). Scale bar equals 10 μm. Data are means ± SD of quadruplicates (^***^*p* < 0.001 vs. untreated control; ^###^*p* < 0.001 vs. WT; Tukey’s multiple comparisons test).

(K-L) U2OS stably expressing RFP-LC3 either WT or knock-in for eIF2α^S51A^ were treated as above for 16 h. After fixation, the cells were stained with a TFE3 antibody followed by an AlexaFluor-488 secondary antibody. Representative images of TFE3 translocation are shown in (K) and the average nuclear intensity of TFE3 was measured (L) Scale bar equals 10 μm. Data are means ± SD of quadruplicates (^***^*p* < 0.001 vs. untreated control; ^#^*p* < 0.05, ^##^*p* < 0.01 vs. WT; Tukey’s multiple comparisons test).

**Figure S2. TFEB and TFE3 regulate ER stress response.**

(A-D) U2OS wild-type or cells knockout for *TFEB* or *TFE3* were treated with tunicamycin (TM, 3 μM), thapsigargin (TG, 3 μM), isobacachalcone (ISO, 25 μM) for 24 h. After fixation, the cells were stained with CHOP-specific antibody followed by an AlexaFluor-568 secondary antibody. Nuclei were counterstained with Hoechst 33342. CHOP nuclear expression was shown in (A, C), and the average nuclear intensity of CHOP was quantified in (B, D). Scale bar equals 10 μm. Data are means ± SD of quadruplicates (^*^*p* < 0.05, ^**^*p* < 0.01, ^***^*p* < 0.001 vs. untreated control; ^#^*p* < 0.05, ^##^*p* < 0.01, ^###^*p* < 0.001 vs. WT; Tukey’s multiple comparisons test).

(E-H) U2OS wild-type or knockout for TFEB or TFE3 cells were treated with 3 μM TM, 3 μM TG or 25 μM ISO for 24 h. After fixation, the cells were stained with ATF4-specific antibody followed by an AlexaFluor-568 secondary antibody. Nuclei were counterstained with Hoechst 33342. ATF4 nuclear expression is shown in (E, G), and the average nuclear intensity of ATF4 was quantified in (F, H). Scale bar equals 10 μm. Data are means ± SD of quadruplicates (^*^*p* < 0.05, ^**^*p* < 0.01, ^***^*p* < 0.001 vs. untreated control; ^#^*p* < 0.05, ^##^*p* < 0.01, ^###^*p* < 0.001 vs. WT; Tukey’s multiple comparisons test).

**Figure S3. ISO** **fails to facilitate the emission of calreticulin and HMGB1.**

(A–D) Human osteosarcoma U2OS cells stably expressing CALR-RFP and HMGB1-GFP were treated with isobacachalcone (ISO, 25 μM) in the presence of low doses of the ICD inducers mitoxantrone (MTX, 1 μM) for 24 h to measure HMGB1 and 8 h to measure calreticulin (CALR). Representative images of CALR-RFP (A) and HMGB1-GFP (C) were shown. The average membrane intensity of CALR-RFP (B) and the intensity of nuclear HMGB1 (D) was assessed by image analysis. Scale bar equals 10 μm. Data are means ± SD of quadruplicates (ns, not statistically significant vs. untreated control, Student’s t-test).
