# Supplementary figures and images for: Isobacachalcone induces autophagy and improves the outcome of immunogenic chemotherapy
Source: Cell Death Dis. 2020 Nov 26;11(11):1015. doi: 10.1038/s41419-020-03226-x (PMC7690654; doi:10.1038/s41419-020-03226-x)

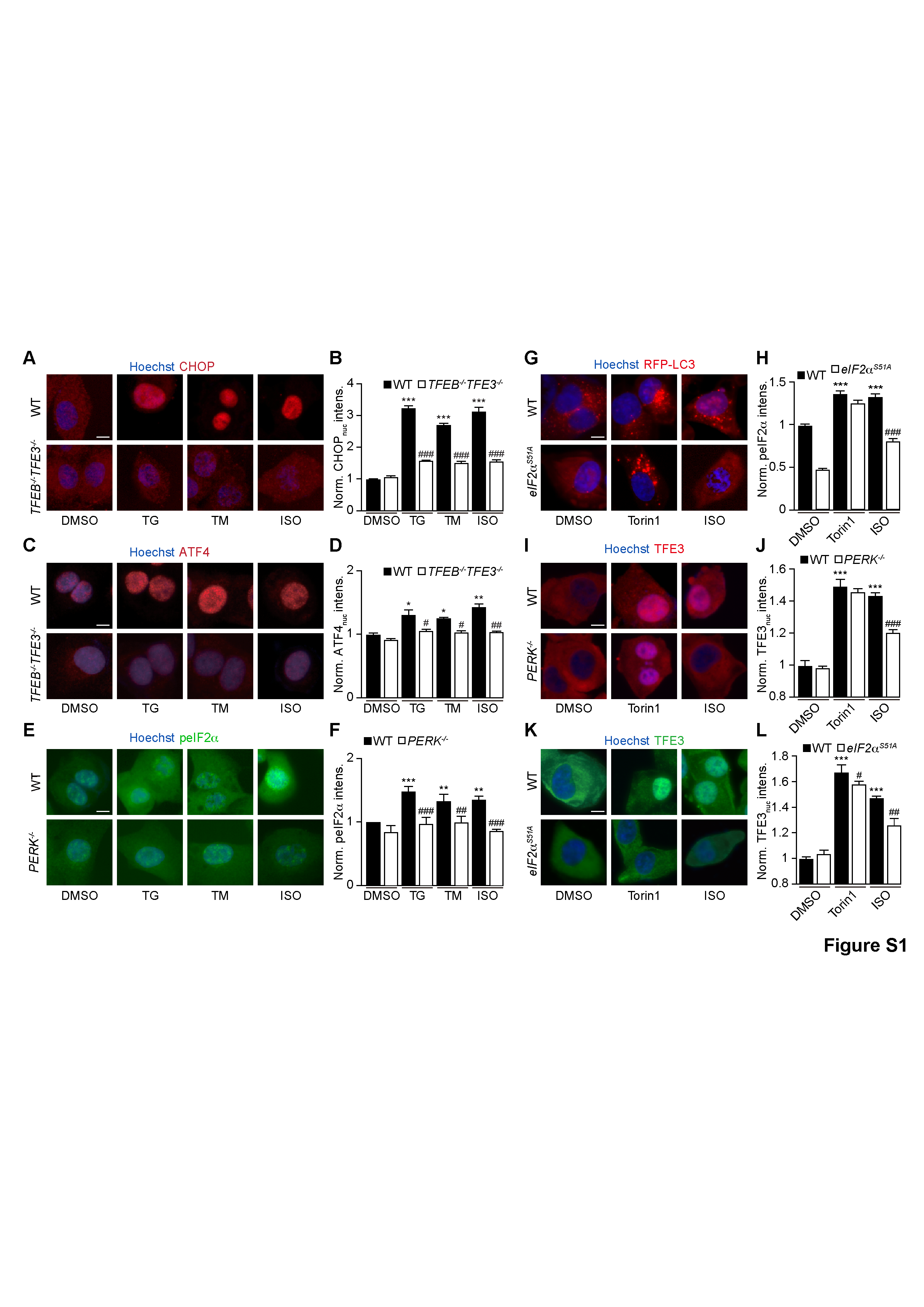

Supplement: Supplementary file 2 — Figure S1 [file 41419_2020_3226_MOESM2_ESM.tif]

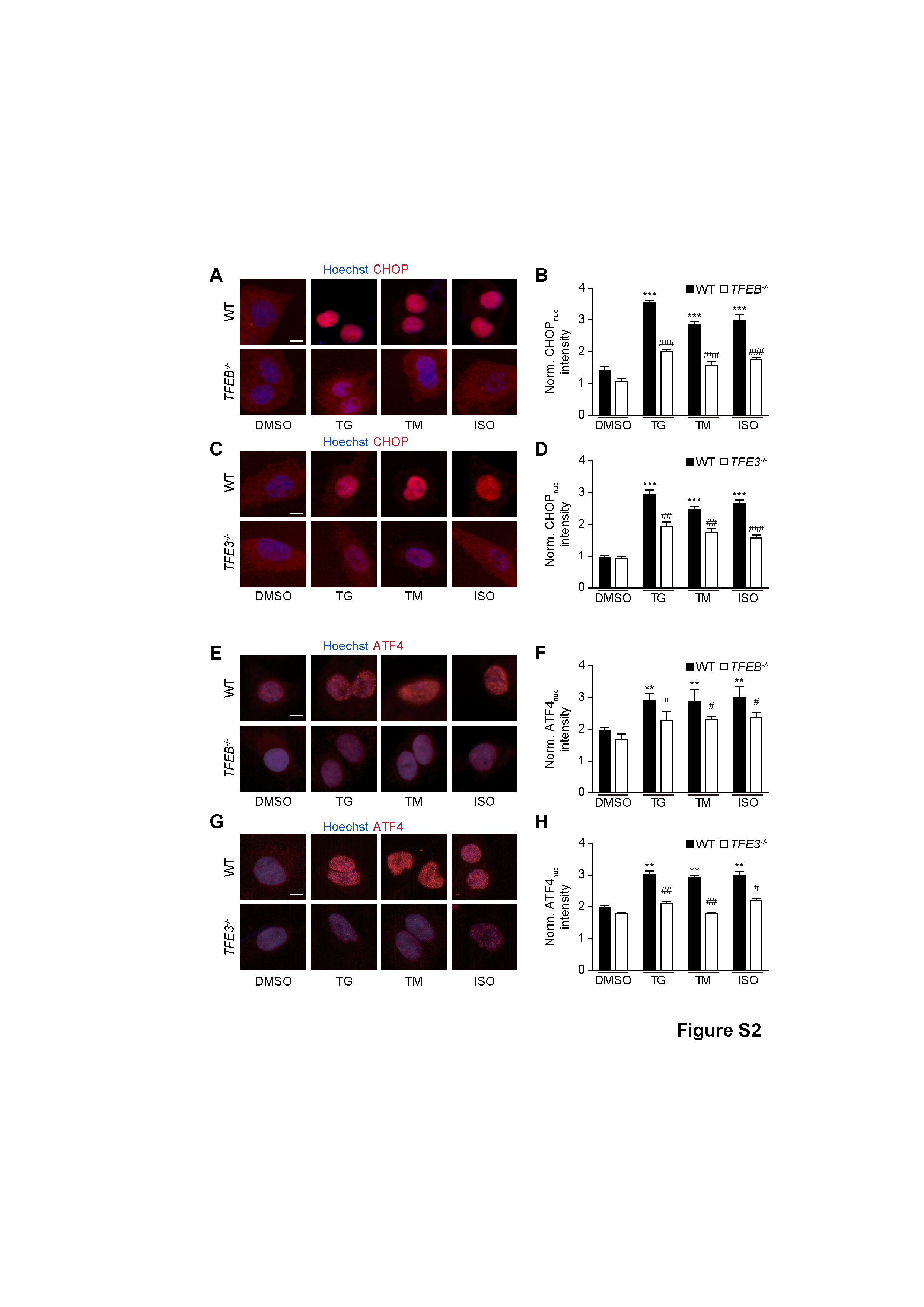

Supplement: Supplementary file 3 — Figure S2 [file 41419_2020_3226_MOESM3_ESM.tif]

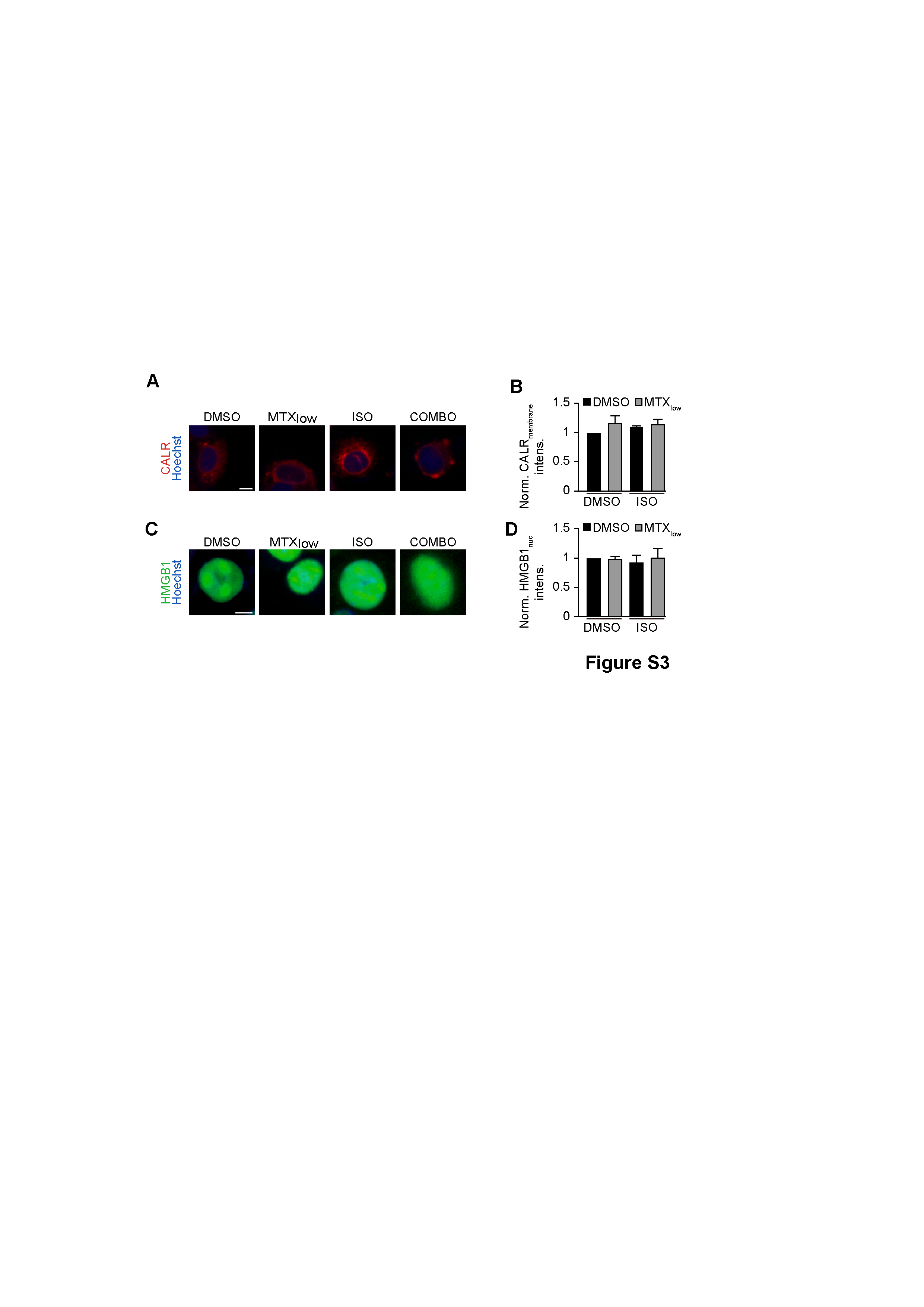

Supplement: Supplementary file 4 — Figure S3 [file 41419_2020_3226_MOESM4_ESM.tif]
